# A previously functional tetracycline-regulated transactivator fails to target gene expression to the bone

**DOI:** 10.1186/1756-0500-4-282

**Published:** 2011-08-11

**Authors:** Eva Schmidt, Maria Eriksson

**Affiliations:** 1 Department of Biosciences and Nutrition, Center for Biosciences, Karolinska Institutet, Karolinska University Hospital, Huddinge, Novum, SE-14183 Stockholm, Sweden

## Abstract

**Background:**

The tetracycline-controlled transactivator system is a powerful tool to control gene expression *in vitro *and to generate consistent and conditional transgenic *in vivo *model organisms. It has been widely used to study gene function and to explore pathological mechanisms involved in human diseases. The system permits the regulation of the expression of a target gene, both temporally and quantitatively, by the application of tetracycline or its derivative, doxycycline. In addition, it offers the possibility to restrict gene expression in a spatial fashion by utilizing tissue-specific promoters to drive the transactivator.

**Findings:**

In this study, we report our problems using a reverse tetracycline-regulated transactivator (rtTA) in a transgenic mouse model system for the bone-specific expression of the Hutchinson-Gilford progeria syndrome mutation. Even though prior studies have been successful utilizing the same rtTA, expression analysis of the transactivator revealed insufficient activity for regulating the transgene expression in our system. The absence of transactivator could not be ascribed to differences in genetic background because mice in a mixed genetic background and in congenic mouse lines showed similar results.

**Conclusions:**

The purpose of this study is to report our negative experience with previously functional transactivator mice, to raise caution in the use of tet-based transgenic mouse lines and to reinforce the need for controls to ensure the stable functionality of generated tetracycline-controlled transactivators over time.

## Introduction

The tetracycline-inducible system (tet-ON/OFF) is a binary transgenic system that enables spatial and temporal regulation of gene expression. It consists of an inducible transcriptional activator (tTA or rtTA) and a tetracycline-responsive promoter element (TRE element, tetop) that controls the transcription of a target gene sequence [1,2]. By placing the sequence of the tTA or rtTA downstream of a tissue-specific promoter, it enables the distinct spatial regulation of expression of a gene of interest inside the mammalian organism [3]. Furthermore, the target gene expression can be regulated quantitatively by exposing the system to tetracycline or its derivative, doxycycline. Temporal control might be of special interest to overcome toxic or lethal reactions from gene products expressed during early development but that one might want to study at later time points [4]. In the tet-OFF system, the tetracycline-controlled activator (tTA) is only active in the absence of doxycycline and binds to the TRE element, which controls the target transgene [5]. In contrast, the reverse tetracycline-controlled activator (rtTA), utilized in the tet-ON system, requires doxycycline to be activated and to bind to the TRE element [6].

Numerous transgenic mouse models have been developed utilizing this system to control gene activity in a broad range of biological systems [7], and more than 100 transactivator (tTA or rtTA) and responsive (pTRE) strains have been published [8]. Our laboratory has generated transgenic mice with different minigenes of wild-type and mutant human lamin A, under the control of the TRE element (tetop), to study the molecular mechanisms underlying Hutchinson-Gilford progeria syndrome (HGPS) [9-11]. The disease is caused by a *de novo *point mutation in exon 11 of the *LMNA *gene (1824C > T, G608G). The mutation results in the activation of a cryptic splice site and an abnormally processed prelamin A protein named progerin [12,13]. HGPS is a rare, segmental progeroid syndrome where the most striking phenotypes affect the skin, the skeletal system and the cardiovascular system. Especially because of the severity of the symptoms of HGPS, the tet system would be useful for studying this disease in transgenic animals. Targeted expression of the lamin A minigene carrying the HGPS mutation (tetop-LA^G608G^) to the skin in mice, using the keratin 5 transactivator (K5tTA) [14], leads to epidermal disease with similar clinical features as have been reported in HGPS patients [10]. To study the skeletal abnormalities in HGPS, we decided to utilize the previously published collagen type 1α1-rtTA (α1p-rtTA) system to create a bone-specific expression model of the HGPS mutation. Even though the transactivator mice had previously been reported to exhibit bone-specific Smad1C expression in the presence of doxycycline [15], when we crossed two founder lines expressing lamin A minigenes, tetop-LA^G608G ^VF1-07 and tetop-LA^WT ^SF1-04, to generate tetop-LA^G608G ^VF1-07; α1p-rtTA and tetop-LA^WT ^SF1-04; α1p-rtTA mice, neither show detectable expression of human lamin A proteins in bone.

## Materials and methods

### Genetically modified mice

α1p-rtTA promoter mice were maintained in mice on C57BL/6J and backcrossed to FVB/NCrl for ten generations to generate FVB/N.Cg-Tg(α1p-rtTA). Transgenic tetop-LA^G608G ^mice, line VF1-07, generated on FVB/NCrl [10], were backcrossed to C57BL/6J for ten generations to generate B6.Cg-Tg(tetop-LA^G608G^,-EGFP)VF1-07. Transgenic tetop-LA^WT ^mice, line SF1-04, generated on FVB/NCrl [10], were maintained on FVB/NCrl. Animals were housed in a 12-hour light/dark cycle at 20-22°C and 55-65% air humidity in a pathogen-free animal facility at the Karolinska University Hospital. The mice were fed irradiated mouse pellets RM3 (Scanbur BK AB). To target HGPS transgenic expression to bone tissue, the mice were intercrossed with α1p-rtTA transgenic mice. To induce transgenic expression, doxycycline (200 μg/ml) in drinking water containing 2.5% sucrose was given during intercross breedings or from postnatal day 0. Offspring from intercross breedings, FVB/N-Tg(tetop-LA^G608G^,-EGFP)VF1-07 and B6.Cg-Tg(α1p-rtTA), and FVB/N-Tg(tetop-LA^WT^,-EGFP)SF1-04 and B6.Cg-Tg(α1p-rtTA), were regarded as 50:50 mixed background (FVB/N;C57BL6). Offspring from intercross breedings, B6.Cg-Tg(tetop-LA^G608G^,-EGFP)VF1-07, and B6.Cg-Tg(α1p-rtTA), and FVB/N-Tg(tetop-LA^G608G^,-EGFP)VF1-07 and FVB/N.Cg-Tg(α1p-rtTA) were regarded as pure background. Offspring from intercrosses between FVB/N-Tg(tetop-LA^G608G^,-EGFP)VF1-07 and additional transactivator lines, B6.Cg-Tg(Sp7-tTA) and B6.Cg-Tg(SM22-rtTA), were used as controls. All mice were genotyped using published protocols [10,15-17]. Mice were born at the expected Mendelian ratios. The animal studies were approved by the Stockholm South Ethical review board, Dnr. S148-03, S141-06 and S107-09.

### RNA and protein extraction

Animals were sacrificed with an overdose of isoflurane (Baxter) at postnatal week 5, 20 or 46, and the long bones, tibia and femur were dissected and the bone marrow removed. The bone tissue was crushed with the Bessmann tissue homogenizer. Bone pieces were transferred to Lysing Matrix D, and Fastprep 220A (Qbiogene) was used twice to further homogenize at 6 m s^-1 ^for 20 seconds. Samples were incubated on ice for 10 minutes between the runs. RNA was extracted using TRIZOL (Invitrogen), and protein was extracted using 8 M Urea/RIPA buffer (containing proteinase inhibitor, Roche). Abdominal aorta was collected from adult tetop-LA^G608G^;SM22-rtTA transactivator mice [16], and mRNA was extracted according to the manufacturer's instructions (Micro Fasttrack™ 2.0 mRNA isolation kit, Invitrogen). To homogenize aorta, Lysing Matrix D and Fastprep 220A was used at 6 m s^-1 ^for 40 seconds.

### RT-PCR and western blot analysis

cDNA was synthesized using random primers according to the instructions for the SuperScript™First-Strand Synthesis System (Invitrogen). RT-PCR for human lamin A and laminAdel150 were performed as previously described [10]. The RT-PCR protocol published by Liu et al. [15] was used to examine the expression of rtTA. PCR amplification of cDNA using primers for β-actin (5'-CCTAGGCACCAGGGTGTGAT-3' and 5'-CCATGTCGTCCCAGTTGGTAA-3') was performed on all samples as a control. Western blot was performed in accordance with previously published procedures [10]. Primary antibodies used for western blot were mouse monoclonal anti-human lamin A+C (mab3211, Chemicon) and mouse monoclonal anti-β-actin (A5441, Sigma).

### Primary osteoblast cultures

The procedure for isolation and culture of primary bone cells from adult mouse calvariae was modified from the protocol published by Bakker and Klein-Nulend [18]. In brief, animals at the age of 5 or 13 weeks were euthanized with an overdose of isoflurane (Baxter). The calvaria was collected and immediately rinsed in phosphate buffered saline (PBS) supplemented with antibiotics (1x AB-AM, Gibco). Bone pieces were cut into smaller fragments and incubated in 2 ml collagenase solution (2 mg/ml Collagenase II, Worthington Biochemical in alpha-MEM M8042, Sigma) and 2 ml 1x Trypsin-EDTA (Gibco) at 37°C on a shaking rotarod. Sequential digestion was performed as described by Bakker and Klein-Nulend [18]. The calvaria pieces were placed in 60-mm culture dishes (Sarstedt) and cultured in 5 ml osteoblast culture medium (alpha-MEM M8042 (Sigma) with 1x AB-AM (Gibco) and 15% heat-inactivated fetal bovine serum (SaveenWerner). The medium was changed every third day until the cell cultures were confluent. Cells were collected using Trypsin-EDTA (Gibco) and a cell scraper (Sarstedt).

### Cytospin

Osteoblasts (1 × 10^3^) were washed and resuspended in 200 μl PBS. Cells were spun on glass slides (SuperFrost Plus, Menzel) using the Shandon Cytospin 4 machine with Shandon filter cards (Thermo) at medium acceleration rate and 500 rpm for 15 minutes. Cells were allowed to dry at RT before they were stored at -8°C.

### Immunofluorescence

Osteoblasts were fixed for 10 minutes in 4% paraformaldehyde at room temperature and permeabilized for 5 minutes using 1% NP-40 in PBS (Pierce). The cells were blocked using 5% serum/0.1% Birj (Pierce) in PBS for 30 minutes [12] and incubated with mouse monoclonal anti-human lamin A+C antibody (mab3211, Chemicon) overnight at 4°C in a 1:5 dilution of blocking solution. On the following day, the cells were washed and incubated with a secondary antibody, anti-mouse Alexa Fluor 594 (1:1000 dilution, Invitrogen). A MIST tray was used for all incubation steps. Cells were mounted using Vectashield mounting media (H-1200, Vectorlab) containing DAPI (4',6-diamidino-2-phenylindole).

## Results and discussion

In this study we have utilized the previously published α1p-rtTA transgenic mice in combination with a transgenic mouse line carrying a minigene of human lamin A with the HGPS point mutation, tetop-LA^G608G^, to create a bone-specific expression model for the disease. A second transgenic mouse line, with only the human wild-type lamin A sequence, tetop-LA^wt^, was used as a control. To circumvent possible problems with genetic background, the congenic strain FVB/N.Cg-Tg(α1p-rtTA) was formed by backcrossing α1p-rtTA for ten generations to FVB/NCrl. For the same purpose, transfer-breeding for tetop-LA^G608G^, line VF1-07, generated on FVB/NCrl, was performed to form congenic strain B6.Cg-Tg(tetop-LA^G608G^,-EGFP)VF1-07. This breeding protocol enabled the analysis of transgenes on both mixed and pure genetic backgrounds.

**Figure 1:**
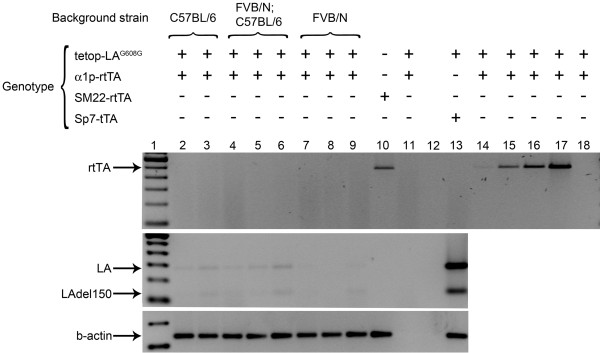
**No detectable expression of transactivator and target gene**. PCR on cDNA from bone (lanes 2-9 and lanes 11-13) and aorta (lane 10) was performed with primer pairs specific for the reverse transactivator. Respective genetic background is indicated for bi-transgenic tetop-LA^G608G+^;α1p-rtTA^+ ^mice. No-reverse transcriptase cDNA sample (lane 11), no-template control (lane 12) and increasing concentrations of genomic DNA template (1, 10, 20, and 50 ng in lanes 14-17, respectively) were included as controls for the PCR assay. PCR on a RNA sample from bone (100 ng of total RNA was used as template for the PCR, lane 18) indicated that there was no significant genomic DNA contamination. Lane 1 is a 100-bp ladder (Invitrogen). Presence and absence of transgene are indicated by + and -, respectively. LA, human lamin A, 276 base pairs; LAdel150, progerin, 123 base pairs. RT-PCR for β-actin served as a control for the RT.

To analyze the expression of the transgene and make sure that the transactivator was present and able to activate the expression of human lamin A in bone, we performed RT-PCR experiments. RT-PCR for human lamin A and lamin Adel150 only showed scant amplification products after 35 cycles of PCR in tetop-LA^G608G+^;α1p-rtTA^+ ^and tetop-LA^wt+^;α1p-rtTA^+ ^(Figure 1 and data not shown). Scant amplification products were also present in samples that did not have the transactivator, tetop-LA^G608G+^;α1p-rtTA^- ^and tetop-LA^wt+^;α1p-rtTA^- ^(data not shown). This result indicated a potential leakiness of the system and is a problem that has been seen by others using this system [19-21]. However, the very low expression could be an effect of inappropriate transcription of the transgene, which might have been due to the fact that the lamin A minigene integration site was active in bone tissue, or an effect of low activity of the transactivator. To directly assess if the transactivator was present in bone tissue, we performed PCR with primers specific for the rtTA on cDNA from bone samples of bi-transgenic animals, tetop-LA^G608G+^;α1p-rtTA^+ ^(Figure 1). We also included cDNA samples from a different rtTA line (SM22-rtTA) [20] as a positive control for the PCR assay (Figure 1). Whereas the positive control sample had a fragment of expected size, there was no amplification in any of the bone samples from α1p-rtTA^+ ^transgenic mice, indicating that the transactivator was not expressed in bone (Figure 1).

Mouse genetic background and transgene integration have been discussed as possible factors for variation in expression levels of transactivator and target gene transcription [23-25]. To test for differences in expression of the transgenes in mice with different genetic background, we performed PCR on cDNA from tetop-LA^G608G+^;α1p-rtTA^+ ^bi-transgenic mice that were on pure C57BL/6, pure FVB/NCrl or mixed C57BL/6;FVB/NCrl background. While the positive control sample, with cDNA from bone of bi-transgenic mice tetop-LA^G608G+^;Sp7-tTA^+^, gave a strong amplification of both the human lamin A and laminAdel 150 transcripts, there was again only very weak amplification in samples from tetop-LA^G608G+^;α1p-rtTA^+ ^bi-transgenic mice on different genetic backgrounds (Figure 1).

Because the PCR assay that analyzed the expression of the transactivator was not cDNA-specific [15] and could amplify genomic DNA, we also included a RNA sample and DNA samples of increasing concentrations from bi-transgenic tetop-LA^G608G+^;α1p-rtTA^+ ^mice, to control for genomic DNA contamination. Amplification was seen for the cDNA sample of tetop-LA^G608G-^;SM22-rtTA^+ ^and in genomic DNA samples that had ≥10 ng of DNA template, but there was no amplification in the RNA sample (Figure 1). This result indicated that there was no transcriptional activity of the α1p-rtTA, independent of genetic background. In addition, the results also indicated that the positive control was an accurate control, even though it amplified genomic DNA, as there was no significant DNA contamination in the RNA extracts and therefore the amplification originated from cDNA.

**Figure 2:**
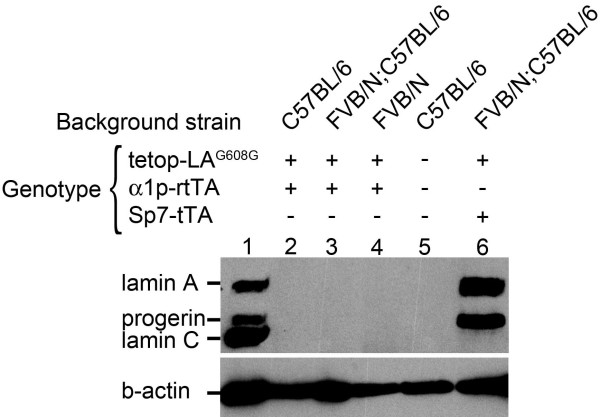
**The α1p-rtTA mouse failed to transactivate the lamin A minigene**. Western blot on bone protein extracts from bi-transgenic animals, tetop-LA^G608G+^;α1p-rtTA^+ ^(lanes 2-4). Human lamin A/C (mab3211) and β-actin (A5441) primary antibodies were used. Protein extracts from a Hutchinson-Gilford progeria sample AG03506 (lane 1) and bone protein extracts from tetop-LA^G608G+^;Sp7-tTA^+ ^mice (lane 6) and wild-type mice (lane 5) were included as positive and negative controls, respectively. Presence and absence of transgene are indicated by + and -, respectively

To analyze the transgenic expression of human lamin A and progerin at the protein level, we performed western blot to screen for transgenic expression using an antibody specific for human lamin A/C (and does not cross-react with mouse lamin A/C) [10,26]. For tetop-LA^G608G+^;α1p-rtTA^+^, bone protein samples were extracted for the different genetic backgrounds, pure FVB/NCrl (n = 6) and C57BL/6J (n = 2) and mixed FVB/NCrl;C57BL/6J (n = 15). For tetop-LA^wt+^;α1p-rtTA^+^, protein samples were extracted from bones of mice on the FVB/NCrl;C57BL/6J background (n = 7). Protein extracts from bone of adult wild-type C57BL/6J, tetop-LA^G608G+^;Sp7-tTA^+^, tetop-LA^G608G-^;Sp7-tTA^+ ^and tetop-LA^G608G-^;Sp7-tTA^- ^mice were included as controls. No lamin A/C protein was detected in any bi-transgenic tetop-LA^G608G+^;α1p-rtTA^+ ^or tetop-LA^wt+^;α1p-rtTA^+ ^or single-transgenic tetop-LA^G608G+^;α1p-rtTA^- ^or tetop-LA^wt+^;α1p-rtTA^- ^mice, showing that the very low levels of RNA transcription detected by RT-PCR were insufficient for protein translation (Figure 2 and data not shown).

**Figure 3:**
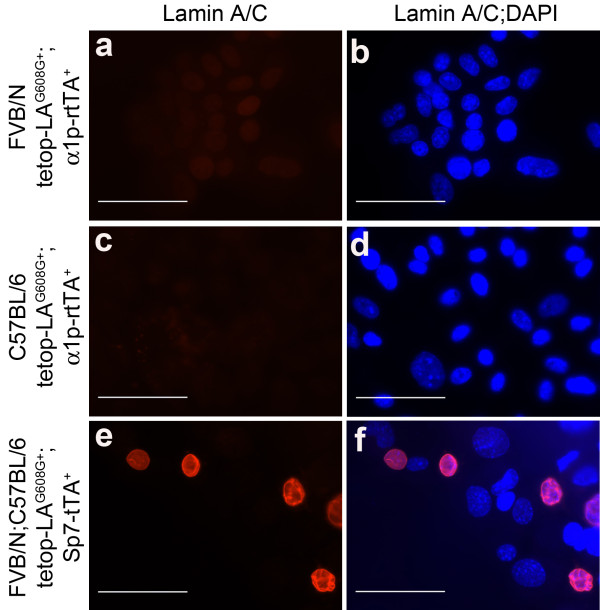
**Lack of the human lamin A minigene protein product in primary osteoblasts from tetop-LA^G608G+^;α1p-rtTA^+ ^bi-transgenic mice**. No immunofluorescence staining was detected when using an antibody specific for human lamin A/C on osteoblasts isolated from bi-transgenic tetop-LA^G608G+^;α1p-rtTA^+ ^mice on different genetic backgrounds, FVB/NCrl **a **and C57BL/6J **c**. Positive staining was obtained using the same antibody on cells from tetop-LA^G608G+^;Sp7-tTA^+ ^bi-transgenic mice **e**. Merged panels with DAPI **b**, **d**, **f**. Scale bars: 50 μm.

In addition to western blot, we analyzed the transgenic expression using immunofluorescence. Primary osteoblasts were isolated from calvariae of 4-week-old bi-transgenic animals (tetop-LA^G608G+^;α1p-rtTA^+^, n = 14) and single-transgenic control mice (tetop-LA^G608G+^;α1p-rtTA^-^, n = 8) on both pure FVB/NCrl and pure C57BL/6J backgrounds. Immunofluorescence staining of cells prepared by cytospin with an antibody specific for human lamin A/C did not detect protein expression in osteoblasts isolated from tetop-LA^G608G+^;α1p-rtTA^+ ^mice, regardless of genetic background (Figure 3a-d). Osteoblasts isolated from tetop-LA^G608G+^;Sp7-tTA^+ ^bi-transgenic mice were included as controls, and osteoblasts from these mice showed staining of the nuclear lamina (Figure 3e-f).

Our expression analysis unequivocally showed that there was no transgenic expression of human lamin A or progerin in bone or osteoblasts from bi-transgenic mice, tetop-LA^G608G+^;α1p-rtTA^+^, and this is most likely the result of a lack of expression of the transactivator. Previous and on-going work in our laboratory, using various target genes of human lamin A in combination with a variety of inducible transcriptional activators for directed expression to multiple tissues (skin, bone, and smooth muscle), have been successful in creating tissue-specific mouse models for HGPS [9, 10, 11 and manuscripts in preparation]. Therefore, we postulate that the reported malfunction of tetop-LA^G608G+^;α1p-rtTA^+ ^as a bone-specific mouse model is due to problems with α1p-rtTA transgenic mice rather than with our human lamin A minigenes. Reasons for the inadequate activity of the transactivator presented in this study remain unclear, and we did not perform any further experiments to investigate the possible underlying mechanisms that might have caused the inactivity of the promoter. However, speculative reasons for defective tetracycline-regulated activators have been discussed before [20,21]. Long-term instability because of epigenetic modification, such as methylation [27,28], problems related to small construct sizes, positional- and dosage-dependent effects [25], and unexpected transgene expression pattern [29] have been reported by various investigators [3,22]. The tet-ON/OFF system is a powerful model system for studying genes *in vitro *or *in vivo*. Previously constructed transactivators are helpful tools within the scientific community that can be easily shared to create new model systems for use in different research contexts. The purpose of this study is to report our negative experience with the α1p-rtTA transgenic mice and to reinforce the obligation of all investigators to monitor the long-term stability of generated tetracycline-controlled transactivators, both over the life-span of an individual mouse and over multiple generations of founder lines.

## Competing interests

The authors declare that they have no competing interests.

## Authors' contributions

ES performed the experiments. ES and ME conceived and designed the study, analyzed the data and wrote the manuscript. All authors read and approved the final manuscript.

## References

[B1] Baron U Bujard H Tet repressor-based system for regulated gene expression in eukaryotic cells: principles and advances Methods Enzymol 2000 327 401 421 11044999 10.1016/s0076-6879(00)27292-3

[B2] Schoenig K Bujard H Generating conditional mouse mutants via tetracycline-controlled gene expression Methods Mol Biol 2003 209 69 104 12357963 10.1385/1-59259-340-2:69

[B3] Furth PA St Onge L Böger H Gruss P Gossen M Kistner A Bujard H Hennighausen L Temporal control of gene expression in transgenic mice by a tetracycline-responsive promoter Proc Natl Acad Sci USA 1994 91 9302 9306 10.1073/pnas.91.20.9302 7937760 PMC44800

[B4] Kistner A Gossen M Zimmermann F Jerecic J Ullmer C Lübbert H Bujard H Doxycycline-mediated quantitative and tissue-specific control of gene expression in transgenic mice Proc Natl Acad Sci USA 1996 93 10933 10938 10.1073/pnas.93.20.10933 8855286 PMC38261

[B5] Gossen M Bujard H Tight control of gene expression in mammalian cells by tetracycline-responsive promoters Proc Natl Acad Sci USA 1992 89 5547 5551 10.1073/pnas.89.12.5547 1319065 PMC49329

[B6] Gossen M Freundlieb S Bender G Müller G Hillen W Bujard H Transcriptional activation by tetracyclines in mammalian cells Science 1995 268 1766 1769 10.1126/science.7792603 7792603

[B7] Tetmouse base http://www.zmg.uni-mainz.de/tetmouse/index.htm

[B8] The Jackson Laboratory: Tet expression systems http://jaxmice.jax.org/research/tet.html

[B9] Hanif M Rosengardten Y Sagelius H Rozell B Eriksson M Differential expression of A-type and B-type lamins during hair cycling PloS ONE 2009 4 e4114 10.1371/journal.pone.0004114 19122810 PMC2606029

[B10] Sagelius H Rosengardten Y Hanif M Erdos MR Rozell B Collins FS Eriksson M Targeted transgenic expression of the mutation causing Hutchinson-Gilford progeria syndrome leads to proliferative and degenerative epidermal disease J Cell Sci 2008 121 969 978 10.1242/jcs.022913 18334552

[B11] Sagelius H Rosengardten Y Schmidt E Sonnabend C Rozell B Eriksson M Reversible phenotype in a mouse model of Hutchinson-Gilford progeria syndrome J Med Genet 2008 45 794 801 10.1136/jmg.2008.060772 18708427

[B12] Eriksson M Brown WT Gordon LB Glynn MW Singer J Scott L Erdos MR Robbins CM Moses TY Berglund P Dutra A Pak E Durkin S Csoka AB Boehnke M Glover TW Collins FS Recurrent de novo point mutations in lamin A cause Hutchinson-Gilford progeria syndrome Nature 2003 423 293 298 10.1038/nature01629 12714972 PMC10540076

[B13] Capell BC Collins FS Human laminopathies: nuclei gone genetically awry Nat Rev Genet 2006 7 940 952 10.1038/nrg1906 17139325

[B14] Diamond I Owolabi T Marco M Lam C Glick A Conditional gene expression in the epidermis of transgenic mice using tetracycline-regulated transactivators tTA and rtTA linked to the keratin 5 promoter J Invest Dermatol 2000 115 788 794 10.1046/j.1523-1747.2000.00144.x 11069615

[B15] Liu Z Shi W Ji X Sun C Jee WS Wu Y Mao Z Nagy TR Li Q Cao X Molecules mimicking Smad1 interacting with Hox stimulate bone formation J Biol Chem 2003 279 11313 11319 14672939 10.1074/jbc.M312731200

[B16] Bernal-Mizrachi C Gates AC Weng S Imamura T Knutsen RH DeSantis P Coleman T Townsend RR Muglia LJ Semenkovich CF Vascular respiratory uncoupling increases blood pressure and atherosclerosis Nature 2005 435 502 506 10.1038/nature03527 15917810

[B17] Rodda SJ McMahon AP Distinct roles for Hedgehog and canonical Wnt signaling in specification, differentiation and maintenance of osteoblast progenitors Development 2006 133 3231 3244 10.1242/dev.02480 16854976

[B18] Bakker A Klein-Nulend J Osteoblast isolation from murine calvariae and long bones Methods Mol Med 2003 80 19 28 12728707 10.1385/1-59259-366-6:19

[B19] Lewandoski M Conditional control of gene expression in the mouse Nat Rev Genet 2001 2 743 755 10.1038/35093537 11584291

[B20] Ryding AD Sharp MG Mullins JJ Conditional transgenic technologies J Endocrinol 2001 171 1 14 10.1677/joe.0.1710001 11572785

[B21] Sun Y Chen X Xiao D Tetracycline-inducible expression systems: New strategies and practices in the transgenic mouse modeling Acta Biochim Biophys Sin 2007 39 235 246 10.1111/j.1745-7270.2007.00258.x 17417678

[B22] Lee S Agah R Xiao M Frutkin AD Kremen M Shi H Dichek DA In vivo expression of a conditional TGF-beta1 transgene: no evidence for TGF-beta1 transgene expression in SM22alpha-tTA transgenic mice J Mol Cell Cardiol 2006 40 148 156 10.1016/j.yjmcc.2005.09.015 16288910 PMC1444940

[B23] Dobie K Mehtali M McClenaghan M Lathe R Variegated gene expression in mice Trends Genet 1997 13 127 130 10.1016/S0168-9525(97)01097-4 9097721

[B24] Opsahl ML McClenaghan M Springbett A Reid S Lathe R Colman A Whitelaw CB Multiple effects of genetic background on variegated transgene expression in mice Genetics 2002 160 1107 1112 11901126 10.1093/genetics/160.3.1107 PMC1462007

[B25] Robertson A Perea J Tolmachova T Thomas PK Huxley C Effects of mouse strain, position of integration and tetracycline analogue on the tetracycline conditional system in transgenic mice Gene 2002 282 65 74 10.1016/S0378-1119(01)00793-4 11814678

[B26] Varga R Eriksson M Erdos MR Olive M Harten I Kolodgie F Capell BC Cheng J Faddah D Perkins S Avallone H San H Qu X Ganesh S Gordon LB Virmani R Wight TN Nabel EG Collins FS Progressive vascular smooth muscle cell defects in a mouse model of Hutchinson-Gilford progeria syndrome Proc Natl Acad Sci USA 2006 103 3250 3255 10.1073/pnas.0600012103 16492728 PMC1413943

[B27] Böger H Gruss P Functional determinants for the tetracycline-dependent transactivator tTA in transgenic mouse embryos Mech Dev 1999 83 141 153 10.1016/S0925-4773(99)00042-8 10381574

[B28] Fedorov LM Tyrsin OY Sakk O Ganscher A Rapp UR Generation dependent reduction of tTA expression in double transgenic NZL-2/tTA(CMV) mice Genesis 2001 31 78 84 10.1002/gene.10007 11668682

[B29] Bao-Cutrona M Moral P Unexpected Expression Pattern of Tetracycline-Regulated Transgenes in Mice Genetics 2009 181 1687 1691 10.1534/genetics.108.097600 19204377 PMC2666531

